# Supplementary material for: Cloud BioLinux: pre-configured and on-demand bioinformatics computing for the genomics community
Source: BMC Bioinformatics. 2012 Mar 19;13:42. doi: 10.1186/1471-2105-13-42 (PMC3372431; doi:10.1186/1471-2105-13-42)
Supplement: Additional file 1 — Supplementary 1 Cloud BioLinux software documentation in the form of a mini, self-contained website. Users need to download and uncompress the .zip file, and open through a web browser the "index.html" file available on the main directory. (ZIP 1823 kb). [file 1471-2105-13-42-S1.ZIP › Cloud-BioLinux-Package-Documentation/docs/mrbayes.html]

Bio-Linux Software Documentation Pages

Back to search form

## mrbayes

|  |  |
| --- | --- |
| Name | mrbayes |
| Description | **MrBayes** is a program for the Bayesian estimation of phylogeny.  Bayesian inference of phylogeny is based upon a quantity called the posterior probability distribution of trees, which is the probability of a tree conditioned on the observations. The conditioning is accomplished using Bayes's theorem. The posterior probability distribution of trees is impossible to calculate analytically; instead, MrBayes uses a simulation technique called Markov chain Monte Carlo (or MCMC) to approximate the posterior probabilities of trees.  MrBayes can be run via the command line by typing: `mrbayes` or `mb` **MrBayes** is available for Bio-Linux in the bio-linux-mrbayes package. A parallel version is available in the bio-linux-mrbayes-multi package. |
| Homepage | http://sourceforge.net/projects/mrbayes/ |
| Remote Documentation | http://mrbayes.csit.fsu.edu/wiki/index.php/Main\_Page |
